# Intercellular mitochondrial transfer as a means of tissue revitalization

**DOI:** 10.1038/s41392-020-00440-z

**Published:** 2021-02-16

**Authors:** Delin Liu, Youshui Gao, Jiao Liu, Yigang Huang, Junhui Yin, Yuyao Feng, Linjing Shi, Bruno P. Meloni, Changqing Zhang, Minghao Zheng, Junjie Gao

**Affiliations:** 1 Centre for Orthopaedic Research, School of Surgery, The University of Western Australia, Nedlands, WA 6009 Australia; 2 Perron Institute for Neurological and Translational Science, Nedlands, WA 6009 Australia; 3 Department of Orthopaedics, Shanghai Jiao Tong University Affiliated Shanghai Sixth People’s Hospital, Shanghai, 200233 China; 4 Department of Hepatobiliary Surgery, Shanghai Public Health Clinical Center, Fudan University, Shanghai, 200083 China; 5 School of Medicine, Zhejiang University, Hangzhou, Zhejiang Province 310058 China; 6 School of Life Sciences, Zhejiang University, Hangzhou, Zhejiang Province 310058 China

**Keywords:** Cell biology, Physiology

## Abstract

As the crucial powerhouse for cell metabolism and tissue survival, the mitochondrion frequently undergoes morphological or positional changes when responding to various stresses and energy demands. In addition to intracellular changes, mitochondria can also be transferred intercellularly. Besides restoring stressed cells and damaged tissues due to mitochondrial dysfunction, the intercellular mitochondrial transfer also occurs under physiological conditions. In this review, the phenomenon of mitochondrial transfer is described according to its function under both physiological and pathological conditions, including tissue homeostasis, damaged tissue repair, tumor progression, and immunoregulation. Then, the mechanisms that contribute to this process are summarized, such as the trigger factors and transfer routes. Furthermore, various perspectives are explored to better understand the mysteries of cell–cell mitochondrial trafficking. In addition, potential therapeutic strategies for mitochondria-targeted application to rescue tissue damage and degeneration, as well as the inhibition of tumor progression, are discussed.

## Introduction

As one of the most complex and important organelles within eukaryotic cells, mitochondria provide essential energy for cell activities. Mitochondrial dysfunction has been shown to be associated with a large number of pathological changes and diseases.^1–3^ Tissues that are energy-consuming or vulnerable to hypoxic–ischemic damage are most likely to be subjected to energy exhaustion due to mitochondrial dysfunction. Thus, maintaining the quantity and quality of mitochondria is critical for tissue homeostasis and cell survival.

For a long time, mitochondria were thought to be constrained within the cytoplasm. Indeed, they undergo frequent reprogramming and intracellular movement.^4^ The bidirectional (anterograde and retrograde) intracellular axonal transport of mitochondria has been widely investigated for its profound effect on mitochondrial homeostasis in neurons.^5,6^ Currently, the critical roles of mitochondrial transfer in tissue homeostasis and development have aroused much interest.^7,8^ In 2004, Rustom et al.^9^ first detected the movement of organelles between mammalian cells via tunneling nanotubes (TNTs), and Spees et al.^10^ demonstrated the intercellular transfer of normal mitochondria from mesenchymal stem cells (MSCs) to mammalian cells with dysfunctional mitochondria in 2006. Since then, accumulating evidence of mitochondrial transfer between cells has revealed that mitochondria are much more active than previously understood,^10–12^ and the transfer of mitochondria from donor cells to recipient cells appears to be a promising approach to realize intercellular energy synchronization.^13–16^

During mitochondrial aerobic respiration, reactive oxygen species (ROS) are also generated as electrons leak from the electron transport chain (ETC). Normally, the number of electrons that escape from the ETC is minimal, and the level of ROS can be controlled via ROS scavenging in the mitochondria.^17^ However, under stress-inducing conditions such as ischemia–hypoxia, chemical exposure, and mitochondrial DNA (mtDNA) deletion, high amounts of ROS produced by enhanced electron leakage accumulate in the mitochondria.^18^ The rapid elevation of ROS levels will dramatically depolarize the mitochondrial membrane potential and subsequently initiate mitophagy, which is a selective autophagic process that degrades damaged mitochondria.^19,20^ Cells cannot survive without this energy supply, thus mitochondrial replacement is undoubtedly an efficient way to revitalize exhausted cells. Intriguingly, an interesting amount of evidence has revealed that mitochondrial transfer occurs in situations besides cell rescue.

Notably, the spontaneous transfer of mitochondria between cells also occurs under physiological conditions during tissue homeostasis and development, which undoubtedly broadens our knowledge of the mitochondrial transfer. On the other hand, under pathological conditions, the intercellular mitochondrial transfer appears to not only rescue tissue damage, which has been frequently reported in the central nervous system (CNS), cardiovascular system, and respiratory system, but also to contribute to multifunctional cellular activity and thereby have an impact on tumor therapy resistance and inflammation regulation. Moreover, the examination of the transcellular degradation of damaged mitochondria from stressed cells also increases our understanding of mitophagy,^21^ and it is compelling to note that stem cells are the most popular donor cells among all the reported transfer cases, indicating that mitochondrial donation might play a pivotal role in stem cell therapy. Here, we summarized the function of the intercellular mitochondrial transfer under both physiological (Table 1) and pathological (Table 2) conditions. We also discuss the potential mechanisms to better understand intercellular mitochondrial communication and provide perspectives on targeted therapy in the future.

**Table 1:** Summary of intercellular mitochondrial transfer under physiological conditions

Donors	Recipients	Induction factor	Transferred cargoes	Route	Transfer outcomes	Ref.
Tissue homeostasis and development						
hMADS	CMs	None	Healthy mitochondria	TNTs	Reprograming of CMs to cardiac progenitor-like cells	^25^
BM-MSCs LT-MSCs BAL-MSCs	BEAS-2B epithelial cells	None	Mitochondria, other cytoplasmic contents	TNTs/MVs/gap junctions	Not verified	^26^
RTCs MMSCs	MMSCs RTCs	None	Mitochondria, cytosol (bidirectional)	TNTs/gap junctions	Induction of MMSC differentiation to kidney tubular cells	^27^
VSMCs BM-MSCs	BM-MSCs VSMCs	None	Healthy mitochondria (bidirectional)	TNTs	Increase in MSC proliferation	^28^
CMs, cardiofibroblasts	Cardiofibroblasts CMs	None	Healthy mitochondria (bidirectional)	TNTs	Structural and functional connectivity for myocardial tissue homeostasis	^29^

**Table 2:** Summary of intercellular mitochondrial transfer under pathological conditions

Donors	Recipients	Induction factor	Transferred cargoes	Route	Transfer outcomes	Ref.
CNS						
Astrocytes	Neurons	Ischemic damage	Healthy mitochondria	MVs (CD38)	Restoration of ATP levels and neuronal viability	^12^
MMSCs	Neurons	Ischemic damage	Healthy mitochondria	TNTs	Recovery of respiration and neurological functions	^31^
MMSCs	Astrocytes	Ischemic damage	Healthy mitochondria	TNTs (Miro1)	Restoration of bioenergetics and promotion of cell proliferation	^32^
EPCs	Brain endothelial cells	OGD	Isolated mitochondria	Internalization	Elevated levels of mitochondrial protein, mtDNA copy number, and intracellular ATP; restoration of endothelial tightness	^35^
Astrocytes	Cerebrospinal fluid	Subarachnoid hemorrhage	Healthy mitochondria	Not verified	Brain recovery and good clinical outcomes	^36^
PC12 cells Soleus cells	Spinal cord-resident cells	SCI	Isolated mitochondria	Internalization	Maintenance of acute bioenergetics after SCI	^37^
BM-MSCs	Motor neurons	OGD/SCI	Healthy mitochondria/isolated mitochondria	TNTs/gap junction	Improved bioenergetics profile and cell survival in post-OGD motor neurons; locomotor functional recovery after SCI	^38^
MSCs	NSCs	Cisplatin	Healthy mitochondria	TNTs (Miro1)	Decrease of NSC death and restoration of mitochondrial membrane potential	^39^
Astrocytes	Neurons	Cisplatin	Healthy mitochondria	Not verified	Increased neuronal survival and restored neuronal calcium dynamics	^40^
HeLa cells	AD mice model	None	Isolated mitochondria	Intravenous injection	Amelioration of cognitive deficits, neuronal loss, and gliosis in Alzheimer’s disease mice	^41^
Astrocytes	Astrocytes	α-Syn ingestion and accumulation	Healthy mitochondria	TNTs	Potential rescue of mitochondrial dysfunction in stressed α-syn-containing astrocytes	^43^
Cardiovascular system						
Pectoralis major muscle cells	CMs	Ischemic damage	Isolated mitochondria	Internalization	Decreased heart infarct size of rabbits	^56^
BM-MSCs	Cardiomyoblasts	Ischemic damage	Healthy mitochondria	TNTs	Rescue of cardioblasts from cell death	^57^
MFs	CMs	Hypoxia/reoxygenation damage	Healthy mitochondria	TNTs	Rescue of CMs from apoptosis	^58^
MSCs	CMs	LPS-induced stress	Healthy mitochondria	TNTs	Enhancement of cardiomyocyte function	^59^
iPSC-MSCs	CMs	Anthracycline-induced cardiomyopathy	Healthy mitochondria	TNTs (TNFαip2, Miro1)	Rescue of CMs damage	^60^
Respiratory system						
BMSCs	Alveolar epithelium cells	LPS-induced ALI	Healthy mitochondria	TNTs/MVs (Cx43)	Restoration of alveolar bioenergetics and elevation of the survival rate	^11^
iPSC-MSCs	Bronchial epithelial cells (BEAS-2B)	CS-induced COPD	Healthy mitochondria	TNTs	Alleviation of CS-induced ATP depletion and lung damage	^61^
MSCs	Lung epithelial cells	Rotenone-induced airway injury	Healthy mitochondria	TNTs (Miro1)	Decrease in apoptosis and lung injury repair	^69^
iPSC-MSCs	Bronchial epithelial cells (BEAS-2B)	Ovalbumin- or CoCl_2_-induced mitochondrial dysfunction	Healthy mitochondria	TNTs (Cx43)	Alleviation of airway inflammation and cell apoptosis	^64^
Musculoskeletal system						
BM-MSCs	Chondrocytes	Rotenone and oligomycin	Healthy mitochondria	Not verified	Not verified	^71^
Osteocytes/MLO-Y4 cell lines	MLO-Y4 ρ° cells	EB-induced mtDNA deletion	Healthy mitochondria	Cell dendrites (ER–mitochondria contact)	ATP reproduction and alleviation of oxidative stress	^73^
Other systems						
BM-MSCs	HUVECs	OGD and reoxygenation	Healthy mitochondria	TNTs	Aerobic respiration and protection of endothelial cells from apoptosis	^74^
Healthy or MCA-treated human MSCs	Injured human MSCs	H_2_O_2_-induced oxidative stress	Healthy mitochondria	TNTs	Decrease of oxidative stress and increase of human MSC viability	^76^
iPSC-MSCs	Corneal epithelial cells	Rotenone-induced mitochondrial dysfunction	Healthy mitochondria	TNTs (TNFαip2)	Protection against oxidative stress and repair of cornea	^75^
WJ-MSCs	MERRF cybrid cells	mtDNA mutation	Healthy mitochondria	TNTs	Reduction of ROS and improvement of mitochondrial bioenergetics	^78^
Tumorigenesis and tumor therapy resistance						
MSC skin fibroblasts	Lung adenocarcinoma A549 ρ° cells	EB-induced mtDNA deletion	Healthy mitochondria/mtDNA	Cytoplasmic extensions or broad cellular contact	Recovery of respiratory function and oxidative metabolism	^10^
Cells in TME of the host mouse	Breast carcinoma 4T1 ρ° cells	EB-induced mtDNA deletion	mtDNA	Not verified	Recovery of respiratory capacity and tumor growth	^94^
MSCs	Melanoma B16 ρ° cells	EB-induced mtDNA deletion	Healthy mitochondria	Not verified	Recovery of respiratory capacity and tumor growth	^95^
hMSCs	Breast cancer MDA-MB-231 cells	None	Isolated mitochondria	Internalization	Increased proliferation and invasion capacities of cancer cells	^93^
BM-MSCs	Osteosarcoma 143B ρ° cells	EB or rhodamine 6G-induced mitochondrial dysfunction	Healthy mitochondria	Not verified	Increase of intracellular ATP levels and restoration of mitochondrial function	^15^
WJ-MSCs	Osteosarcoma 143B ρ° cells	EB-induced mtDNA deletion	Healthy mitochondria	TNTs	Restoration of bioenergetics and OXPHOS-dependent cellular growth	^77^
Mesothelioma cells	Mesothelioma cells	Acidified hyperglycemic medium	Vesicles, mitochondria, proteins—bidirectional	TNTs	Cancer cell pathogenesis and invasion	^83^
T24 bladder cancer cells	RT4 bladder cancer cells—noninvasive	None	Healthy mitochondria	TNTs	Increased invasiveness of RT4 cells	^98^
BMSCs	Primary AML blasts	None	Healthy mitochondria	TNTs (NOX2, ROS)	Energy acquirements of the proliferating cancer cells	^89^
	Nonmalignant CD34^+^ cells	H_2_O_2_		Not verified	Not clarified	
Highly glycolytic CAFs	Prostate cancer cells	None	Healthy mitochondria	TNTs	Increased OXPHOS metabolism and respiratory capacity of cancer cells	^97^
Neighboring nonmalignant BMSCs	Multiple myeloma cells	None	Healthy mitochondria	TNTs (CD38, direct contact)	Increased ATP production and mitochondrial respiration of multiple myeloma cells	^84^
Endothelial cells, MSCs	MCF7 breast cancer cells	Doxorubicin	Healthy mitochondria	TNTs	Chemoresistance	^92^
Healthy PC12 cells	Pheochromocytoma-derived PC12 cells	UV radiation	Healthy mitochondria	TNTs	Rescue of apoptotic P12 cells	^87^
Astrocytoma cells	Astrocytoma cells	Radiation treatment	Mitochondria, Calcium	Tumor microtubules (Cx43)	Formation of a self-repairing and radio-resistant network	^85^
U87 glioblastoma cells	Adjacent U87 glioblastoma cells	Etoposide	Mitochondria	TNTs	Perinuclear concentration and rearrangement of mitochondria	^86^
Bone marrow-derived MS-5 cell line	AML cells	Cytarabine	Healthy mitochondria	Endocytosis	Increase of ATP production and survival capacity of AML cells following chemotherapy	^88^
Chemoresistant ovarian cancer cells	Chemoresistant or chemosensitive ovarian cancer cells	Hypoxia	Mitochondria	TNTs (mTOR pathway)	Synchronization of cancer cells against drug therapy	^96^
Immunoregulation						
BM-MSCs	Monocyte-derived macrophages	LPS-induced ARDS	Healthy mitochondria	TNTs	Enhance the phagocytic effect of macrophages	^62^
BM-MSCs	Monocyte-derived macrophages	Escherichia coli infection	Healthy mitochondria	TNTs	Enhance the oxidative phosphorylation and phagocytosis of macrophages	^104^
BM-MSCs	Macrophages	LPS-induced ARDS	Healthy mitochondria	MVs	Promote an anti-inflammatory and highly phagocytic phenotype of macrophagy and ameliorate lung injury	^63^
BM-MSCs	Th17 cells	IL-2	Healthy mitochondria	Cell contact-dependent manner	Increased oxygen consumption and reduced production of pro-inflammatary cytokines; generation of T-regulatory cells	^105^
MSCs	PBMCs	None	Healthy mitochondria	Not verified	T cell activation and T-regulatory (Treg) cell differentiation; suppressive effect of inflammatory response	^106^
	CD3^+^ T cells		Isolated mitochondria	Internalization		
Intercellular degradation						
Retinal ganglion cells	Adjacent astrocytes	Rotenone	Damaged mitochondria	MVs	Donors: mitophagy for transcellular degradation and self-protection	^21^
BM-MSCs	Macrophages	Oxidative stress to MSCs	Depolarized mitochondria	MVs	Donors: Mitophagy of MSCs for self-protection Recipients: enhancement of macrophage energetics	^107^
T-ALL cells	BM-MSCs	Ara-C- or MTX-induced intracellular oxidative stress	Damaged mitochondria	TNTs	Donors: mitophagy for reduction of intracellular ROS and enhancement of chemoresistant capacity	^91^
BM-MSCs	T-ALL cells	Ara-C- or MTX-induced intracellular oxidative stress	Healthy mitochondria	TNTs	Recipients: chemoresistance	^91^
Stressed CMs or HUVECs	MSCs	H_2_O_2_-induced oxidative stress	Damaged mitochondria	TNTs (ROS)	Donors: transmitophagy of stressed cells	^113^
MSCs	Stressed CMs or HUVECs	H_2_O_2_-induced oxidative stress	Healthy mitochondria	TNTs (HO-1)	Recipients: survival of stressed cells	^113^

## Mitochondrial transfer for physiological tissue homeostasis and development

Cell therapy, particularly that based on stem cells, has been deemed as a prospective approach to repair cardiac diseases,^22–24^ but the specific intercellular signaling mechanisms are still obscured. To further investigate the impact of the cross-talk between cells, Acquistapace et al.^25^ cocultured fully differentiated mouse cardiomyocytes (CMs) with hMADs (human multipotent adipose-derived stem cells), and first revealed the essential function of mitochondrial transfer from stem cells to CMs for somatic reprogramming. The proportion of cardiac progenitor-like cells was dramatically decreased after mtDNA in stem cells was depleted. Considering that MSCs isolated from specific tissues show subtle heterogeneity, Sinclair et al.^26^ compared the efficacy of mitochondrial transfer between bone marrow-mesenchymal stem cells (BM-MSCs) and two other populations of MSCs derived from healthy lung tissues (LT-MSCs) and bronchoalveolar lavage fluid of lung transplant recipients (BAL-MSCs) in vitro. The results indicated that LT-MSCs and BAL-MSCs can also donate cytoplasmic content and mitochondria spontaneously to healthy human bronchial epithelial cells with a similar efficiency via unidirectional transfer.

Notably, several in vitro studies found that this spontaneous intercellular transfer of mitochondria could also be bidirectional. Intercellular exchanges of the cytoplasm and mitochondria between RTCs (renal tubular cells) and mesenchymal multipotent stromal cells (MMSCs) were detected within a coculture system and these were also bidirectional, although the transport towards MMSCs was predominant.^27^ It is plausible that the bidirectional exchange of cellular components probably contributes to differentiation of MMSCs, as the expression of renal tubule-specific proteins was observed in MMSCs.^27^ Similarly, equivalent bidirectional exchange of mitochondria was detected under normal culturing conditions between human VSMCs (vascular smooth muscle cells) and BM-MSCs, and this process promoted MSC proliferation but not differentiation.^28^ On the other hand, the spontaneous bidirectional mitochondrial transfer also occurs between somatic cells via nanotubes, as evidenced by the intercellular communication between CMs and cardiofibroblasts, which provides structural and functional connectivity for myocardial tissue homeostasis.^29^ Although studies of intercellular mitochondrial transfer that occur without stress factors are limited (Table 1), it is still a meaningful aspect that should be noted to investigate its potential role in maintaining tissue homeostasis.

## Mitochondrial transfer under pathological conditions

### Mitochondrial transfer in the CNS (Table 2)

Bidirectional mitochondrial transport within neuronal axons is a distinctive intracellular activity necessary to meet dynamic energy needs in different regions of neurons.^6^ Recently, intercellular mitochondrial transfer has also been shown to be a nonnegligible biological event in the CNS and is thought to play crucial roles continuously in ischemic and hemorrhagic damage rescue,^12,30–36^ spinal cord injury (SCI) recovery,^37,38^ neuronal protection of neurons from chemotherapy-induced neurotoxicity,^39,40^ and neurodegeneration.^41–43^

A study involving a mouse model of stroke verified that functional mitochondria in astrocytes can be delivered to damaged neurons for the purpose of ischemic injury repair and neurorecovery.^12^ This intercellular transfer of mitochondria is likely mediated by a calcium-dependent mechanism involving CD38 signaling, and suppression of CD38 signaling may result in a reduction in transferred mitochondria, cell viability, and post-stroke recovery.^12^ Babenko et al.^31,32^ showed that mitochondria from multipotent MSCs can be transferred to neurons or astrocytes, leading to the restoration of respiration in recipient cells and the alleviation of ischemic damage. Apart from MSCs, endothelial progenitor cells (EPCs) have also been used for cell therapy because of their ability to regulate angiogenesis and vasculogenesis.^33,34^ Hayakawa et al.^35^ confirmed that EPC-originating extracellular mitochondria can be delivered into damaged brain endothelial cells (ECs). Their results showed that the levels of the mitochondrial protein TOM40, the mtDNA copy number, and ATP production were all elevated in damaged brain ECs. Endothelial tightness was restored after the treatment with EPC-derived mitochondrial particles, showing that EPC-derived mitochondria may support the function of brain ECs. In addition, studies concerning the translocation of mitochondria after subarachnoid hemorrhage (SAH) and SCI have also been reported. Chou et al.^36^ researched both a rat model and human patients with and without SAH. The results showed that the mitochondria of astrocytes can be transferred to cerebrospinal fluid (CSF) after SAH. Moreover, the extracellular mitochondrial membrane potentials appeared to be reduced within the first 72 h after SAH and started to increase thereafter, which was also consistent with the occurrence of poor and good clinical outcomes after SAH, respectively. A novel experiment concerning SCI demonstrated that exogenous mitochondria could be transplanted into the injured rat spinal cord and contribute to the maintenance of acute bioenergetics as well as functional recovery after SCI, even though long-term functional neuroprotection did not ultimately occur.^37^ In another coculture system, mitochondria derived from BM-MSCs could be transferred to oxygen glucose-deprived neurons and improve the survival of motor neurons after oxygen glucose deprivation (OGD), which illustrated the potential therapeutic effect of the mitochondria on SCI.^38^ Further study showed that both transplantation of BM-MSCs and mitochondria derived from BM-MSCs could decrease neuronal apoptosis and promote locomotor functional recovery in SCI rats, indicating that mitochondrial transfer might be a potential mechanism of stem cell therapy in SCI.^38^

Cognitive deficits induced by chemotherapy is one of the critical concerns for cancer treatment.^44,45^ It has been demonstrated that cisplatin, a platinum-based chemotherapeutic agent, can disrupt synaptosomal mitochondrial function and change neuronal mitochondrial morphology in mice.^46^ Recently, Heijnen’s team reported the protective effects of intercellular mitochondrial transfer on cisplatin-induced neurotoxicity.^39,40^ In a coculture system, MSCs transferred their healthy mitochondria to cisplatin-treated neural stem cells (NSCs), resulting in a decrease in NSC death and restoration of the mitochondrial membrane potential.^39^ Furthermore, they verified that transfer of astrocyte-derived mitochondria to damaged neurons induced by cisplatin in vitro can increase neuronal survival and restore neuronal calcium dynamics.^40^ Intriguingly, the same dose of cisplatin in astrocytes did not affect astrocyte viability.^40^ The results indicated that astrocytes might protect neurons from chemotherapy-induced neurotoxicity in vivo by donating their healthy mitochondria to damaged neurons.

Mitochondrial dysfunction is an important component of neurodegenerative diseases such as Alzheimer’s disease (AD) and Parkinson’s disease (PD).^41,42,47,48^ An investigation revealed that AD mice treated intravenously with freshly isolated human mitochondria showed better cognitive performance than the mice in the control group, and that a substantial decrease in neuronal loss and gliosis was observed in mitochondria-treated mice compared to that in untreated AD mice.^41^ Although the efficacy and safety concerns need to be further considered, this research provides a potential therapeutic target for improving mitochondrial biogenesis in AD patients. The progression of PD is related to aggregation of pathological α-synuclein (α-syn).^49^ Recent evidence suggests that pathological α-syn aggregations could bind to the mitochondria with high affinity and subsequently lead to mitochondrial toxicity and dysfunction.^50^ Interestingly, Rostami et al.^43^ revealed that α-syn could pathologically accumulate in stressed astrocytes, which resulted in swelling of the endoplasmic reticulum (ER) and impaired mitochondrial dynamics. Furthermore, excess α-syn in stressed astrocytes was delivered to adjacent healthy astrocytes via direct contact or TNTs, which in turn induced the transfer of mitochondria from healthy astrocytes to stressed astrocytes.^43^ The transfer of pathological α-syn between cells and the role of intercellular mitochondrial transfer in PD progression suggests a therapeutic target for the treatment of PD in the brain.

### Mitochondrial transfer in the cardiovascular system (Table 2)

The heart is a highly energetic and autonomic organ that requires a continuous oxygen supply to maintain its physiological function. Mitochondria provide the primary energy for the heart by aerobic respiration and constitute 30% of the volume of CMs.^51^ Thus, cardiovascular mitochondrial dysfunction or mtDNA mutations induced by increased oxidative and nitro-oxidative stress are closely associated with cardiovascular diseases.^3,52,53^

Ischemia is a major cause of myocardial damage and apoptosis because blocking the oxygen supply to CMs generally leads to mitochondrial dysfunction.^3,54,55^ It has been demonstrated that the transplantation of autologous pectoralis-derived functional mitochondria to ischemic myocardial tissue resulted in apparent cardioprotection, and greatly decreased infarct size of the heart after 4 weeks of recovery in rabbits.^56^ By using the fluorescence imaging, mitochondria were observed to be partly internalized by CMs 2–8 h after transplantation. Although the specific mechanism of mitochondrial internalization was not revealed, the results showed that the transplanted mitochondria could enhance oxygen consumption, ATP production and chemokine secretion within the ischemic myocardial tissue, and also promote the expression of protein pathways that are important in preserving myocardial energetics.^56^ In addition to direct mitochondrial transplantation, MSCs also exhibit the potential to rescue ischemia-exposed cardiomyoblasts from cell death by mitochondrial donation in the coculture system.^57^ In another hypoxia/reoxygenation injury model of CMs, unidirectional mitochondrial transfer, either from intact or hypoxia/reoxygenation-treated myofibroblasts to damaged CMs, was detected to attenuate CM apoptosis.^58^ The results updated their previous study on intercellular mitochondrial transfer, revealing the bidirectional transfer of mitochondria between cardiofibroblasts and CMs under normoxia.^29^ In addition, damaged CMs induced by lipopolysaccharide (LPS)^59^ or anthracycline^60^ can also be rescued by functional mitochondria derived from MSCs.

### Mitochondrial transfer in the respiratory system (Table 2)

Intercellular mitochondrial transfer from MSCs to recipient cells also occurs when the respiratory system is exposed to the risk of injury or inflammation.^11,61–64^ Exogenously purified BMSCs showed a protective effect when they were administered in a model of sepsis-induced acute lung injury (ALI), suggesting that BMSCs might show therapeutic benefits for damage repair.^65^ To further explore the protective mechanism of BMSCs, Islam et al.^11^ first monitored BMSC-derived mitochondria in the alveolar epithelium of an LPS-induced ALI mouse model. After the injection of human BMSCs, human mtDNA was found in mouse lungs. Specifically, the transferred functional mitochondria increased the ATP level in alveolar cells and the secretion of surfactant from alveolar type II (AT2) cells, which restored alveolar bioenergetics and elevated the mouse survival rate. Cigarette smoke (CS) exposure has been verified to cause mitochondrial dysfunction and to be harmful to energy metabolism in mouse lungs.^66^ In a CS-induced chronic obstructive pulmonary disease rat model, induced pluripotent stem cell-derived MSCs (iPSC-MSCs) also presented active mitochondrial transfer capacity, which contributed to the rescue of damaged mitochondria in adjacent airway epithelial cells with elevated ATP levels, while BM-MSCs exhibited a lower capacity for damage rescue when compared to iPSC-MSCs.^61^ This may be due to the stronger capacity of iPSC-MSCs to proliferate and the increased time length of differentiation potential maintenance.^67,68^ In a rotenone-induced airway injury model, Ahmad et al.^69^ first observed that the overexpression of Miro1 (mitochondrial Rho-GTPase 1), a calcium-sensitive adaptor protein that promotes intercellular mitochondrial transfer from BM-MSCs to lung epithelial cells, increased the rescue efficacy of stem cells and reduced airway hyperresponsiveness. Similarly, mitochondrial transfer from iPSC-MSCs to epithelial cells was also shown to reduce mitochondrial dysfunction and alleviate airway inflammation in a mouse model of asthma.^64^

### Mitochondrial transfer in the musculoskeletal system (Table 2)

Studies on the intercellular mitochondrial transfer in the musculoskeletal system have mainly focused on chondrocytes and osteocytes. In chondrocytes, mitochondrial dysfunction occurs immediately following cartilage injury, and leads to chondrocyte death, cartilage degeneration, and ultimately post-traumatic osteoarthritis.^70^ When exposed to mitochondria-specific stressors, such as rotenone and oligomycin, chondrocytes will receive mitochondria from MSCs in the coculture system.^71^ Although the function of such mitochondrial donation has not yet been elucidated, it is expected that mitochondrial rescue could attenuate the chondrogenic stress.

Osteocytes embedded in the bone matrix are connected by dendritic networks and coordinate the function of osteoblasts and osteoclasts.^72^ Thus, it is critical to maintain the survival of osteocytes under deleterious conditions, including mechanical loading, hormone stimulation, and other stressors, during their long lifespan of up to 40 years.^72^ Recently, our group verified that the distribution of mitochondria in primary osteocyte dendrites decreased with aging.^73^ Furthermore, we found that intercellular mitochondrial transfer also occurs within the osteocyte dendritic network and that the transferred mitochondria restored the cellular metabolism in stressed osteocytes without functional mitochondria.^73^ In addition, we found that ER–mitochondria contact plays a crucial role in mediating the transfer of mitochondria between osteocytes.^73^ Intriguingly, mitofusion 2 (Mfn2), a tethering protein involved in ER–mitochondria contact, was also reduced in aging osteocytes,^73^ which might impair the transfer of mitochondria between aged osteocytes. Thus, it is highly plausible that mitochondrial transfer within the osteocyte dendritic network participates in maintaining the viability of osteocytes and regulating the bone homeostasis during aging.

### Mitochondrial transfer in other tissue systems (Table 2)

MSCs can originate from various sources, and different types of MSCs have different potentials for somatic differentiation and cell rescue. In addition to the effect on the rescue of the most vulnerable organs mentioned above, MSCs were also observed to transfer mitochondria to other stressed cells and restore their aerobic respiration. Human umbilical vein EC that were damaged by OGD and reoxygenation could be rescued by culture with human BM-MSCs, and this effect was mediated by the unidirectional transfer of healthy mitochondria from BM-MSCs to injured ECs.^74^ In another coculture system, mitochondrial transfer from human iPSC-MSCs to rabbit corneal epithelial cells (CECs) was detected under native conditions, and the transfer efficiency was greatly enhanced in CECs under rotenone-induced oxidative stress.^75^ The transplantation of a scaffold containing healthy MSCs enhanced corneal wound healing in a rabbit model of alkali-induced injury, and the transfer of MSC-derived mitochondria was detected in corneal epithelium.^75^ Mitochondrial transfer also occurred between the same type of human MSCs when the recipient MSCs were overoxidized by H_2_O_2_, and the donor MSCs were pretreated with *N*-acetyl-l-cysteine (NAC) and l-ascorbic acid 2-phosphate, which had been demonstrated to be beneficial for mitochondrial biogenesis in MSCs.^76^ Consequently, the oxidative stress was decreased in H_2_O_2_-treated MSCs and the fragmentation of damaged mitochondria was also alleviated.^76^ Recently, human umbilical cord Wharton’s jelly mesenchymal stem cells (WJ-MSCs) have been shown to be another good source of donor mitochondria.^77^ Myoclonus epilepsy with ragged-red fiber (MERRF) syndrome is a representative mitochondrial disease that leads to neuromuscular disorders. Coculture of WJ-MSCs with MERRF cybrid cells that exhibited a high mitochondrial mutation ratio improved mitochondrial bioenergetics and viability of MERRF cybrids.^78^ This rescue effect was also attributed to the transfer of healthy mitochondria from WJ-MSCs to MERRF cybrid cells.

### Mitochondrial transfer during tumorigenesis and tumor therapy resistance (Table 2)

It has been increasingly recognized that tumors do not behave as simple masses of malignant cells, but rather as complex mixtures of tumor cells and non-tumor cells from the tumor microenvironment (TME) that undergo dynamic progression involving resistance to different stimuli and therapies.^79–81^ Mitochondrial transfer between tumor cells and other cells within the TME is also an important biological behavior of cancer involved in respiration recovery, tumorigenesis, and therapy resistance. The self-protective transfer of mitochondria has been reported in many different tumor settings, including melanoma,^82^ malignant pleural mesothelioma,^83^ multiple myeloma,^84^ osteosarcoma,^15,77^ astrocytoma,^85^ glioblastoma,^86^ pheochromocytoma,^87^ acute myeloid leukemia (AML),^88–90^ acute lymphoblastic leukemia (ALL),^91^ and in lung,^10^ breast,^92–95^ ovarian,^92,96^ prostate,^97^ and bladder^98^ cancers.

Spees et al.^10^ exposed the human lung adenocarcinoma-derived A549 cell line to ethidium bromide to induce mtDNA deletion and the cells consequently became incapable of aerobic respiration and growth (A549 ρ° cells). Surprisingly, A549 ρ° cells were shown to acquire functional mtDNA and mitochondria after coculture with human MSCs or skin fibroblasts and consequently regained their respiratory function and capacity for oxidative metabolism. Berridge and Tan^82^ demonstrated that the tumorigenicity of metastatic murine melanoma (B16) and breast carcinoma (4T1)^94^ ρ° cells without mtDNA was lagged behind that of parental tumor cells, and this was proposed to be mainly caused by the absence of mitochondrial respiratory function. However, ρ° cells regained mtDNA from the TME of the host mouse, which resulted in the recovery of oxidative phosphorylation (OXPHOS) and tumor growth.^94^ The acquisition of mtDNA by ρ° cells was later shown to be involved in whole mitochondrial transfer from MSCs during coculture with ρ° cells.^95^ This series of studies revealed the essential effect of mitochondrial respiration on tumor formation, as B16 ρ° cells do not form tumors unless they acquire mtDNA.^95^ Inhibition of either complex I- or complex II-dependent respiration leads to impaired tumorigenicity.^95^ Another study also claimed that MSC-derived mitochondria increased the proliferation and invasion capacities of MDA-MB-231 breast cancer cells, accompanied by enhanced OXPHOS activity and ATP production in cancer cells.^93^ In solid cancers, cancer-associated fibroblasts (CAFs) engage in tumor progression by reprogramming the metabolism of cancer cells.^99^ A recent study suggested that highly glycolytic CAFs tend to donate their dispensable mitochondria to adjacent prostate cancer cells, resulting in enhanced OXPHOS metabolism and the respiratory capacity of cancer cells.^97^ It is plausible that the recruitment of mitochondria from CAFs is another pathway allowing high energy-consuming malignant cells to enhance their intracellular metabolism, which might contribute to their enhanced malignancy. Although respiration restoration is an indispensable element for tumorigenesis of ρ° cancer cells, it is unclear which process of OXPHOS activity is the key event for tumor growth. Noteworthy, a recent study clearly documents for breast cancer and melanoma that the major reason for respiration restoration in ρ° cancer cells is to drive dihydroorotate dehydrogenase (DHODH)-dependent respiration that is essential for de novo pyrimidine synthesis, not for ATP formation.^100^ Deletion of DHODH in cancer cells with fully functional OXPHOS significantly inhibited tumor formation, while supression of mitochondrial ATP synthase has little effect.^100^ The results indicated that DHODH activation and coenzyme Q redox cycling during the electron transport of functional OXPHOS activity is essential for tumorigenesis, suggesting DHODH as a potential broad-spectrum target for cancer therapy.^100^

Therapy resistance in cancer is still an important issue for ensuring the effectiveness of therapy. Many studies have reported potential underlying mechanisms, including intrinsic and extrinsic processes, and the extrinsic processes are influenced greatly by intratumoral heterogeneity.^101^ Specifically, one significant factor that leads to intratumoral heterogeneity is that the TME contains many nonmalignant cells, including CAFs, MSCs, and immune cells that are recruited to the tumor site.^80,102^ Intercellular communications and interactions between malignant cells and nonmalignant cells have been shown to play a significant role in tumor heterogeneity and drug resistance.^101^ Pasquier et al.^92^ showed that mitochondrial transfer from ECs to MCF7 breast cancer cells increased their resistance to chemotherapy and that transfer of mitochondria preferentially occurred between ECs and cancer cells rather than MSCs and cancer cells in a tri-culture system. However, the mechanism of this selective mitochondrial transfer has not been precisely described. Pheochromocytoma-derived PC12 cells exposed to ultraviolet light radiation were reported to acquire functional mitochondria from healthy PC12 cells in the coculture system, which protected those stressed PC12 cells against apoptosis.^87^ Similar biological behavior was also observed in tumors of the CNS.^85,86^ Astrocytoma cells tend to interconnect with adjacent cells to form a functional, radio-resistant network through which mitochondria can be transferred between cells.^85^ Another study of U87 glioblastoma cells detected the perinuclear accumulation of mitochondria after the cells were treated with etoposide and recognized that cell-to-cell transfer of mitochondria may contribute to resistance to anticancer therapy.^86^ Among the hematopoietic cancer cell lines, AML cells were verified in vitro or in vivo to uptake functional mitochondria from surrounding bone marrow stromal cells, leading to an increase in ATP production in recipient AML cells and a downward trend of mitochondrial depolarization after drug treatment, which was responsible for chemotherapy resistance.^88^ Marlein et al.^89^ verified the transfer of mitochondria from BMSCs to AML blasts, and first reported that the elevation of NADPH oxidase-2-derived oxidative stress in AML cells drives the transfer of functional mitochondria, contributing to the energy requirements of rapidly proliferating cancer cells.

### Mitochondrial transfer involved in immunoregulation (Table 2)

During the process of tissue repair, macrophages play a critical role by clearing inflammatory products through phagocytosis. MSCs can enhance the anti-inflammatory capacity of macrophages by inducing the differentiation of M2 phenotype macrophages.^103^ A series of in vitro and in vivo studies showed that mitochondria transferred from MSCs to macrophages may drive the selective differentiation of macrophages towards anti-inflammatory M2 phenotype and contribute to the antimicrobial effect of MSCs.^62,63,104^ In an acute respiratory distress syndrome environment, the OXPHOS activity and phagocytosis of macrophages was increased after they received healthy mitochondria from MSCs,^62,104^ and enhanced OXPHOS was thought to be responsible for the M2 phenotype conversion of macrophages.^63^ In turn, inhibition of intercellular mitochondrial transfer either by damaging mitochondria of MSCs^63^ or blocking the route of transfer^62,104^ failed to improve the phagocytosis and bioenergetics of macrophages. In addition to macrophages, pathogenic T helper 17 (Th17) cells also tended to acquire mitochondria from BM-MSCs in a coculture system, which increased oxygen consumption and reduced IL-17 production by Th17 cells.^105^ Moreover, in rheumatoid arthritis patients, reduced mitochondrial transfer to Th17 cells was observed in synovial stromal stem cells compared with that in BM-MSCs isolated from healthy donors.^105^ In a new study, the effect of cell–cell transfer and direct transplantation of mitochondria derived from MSCs to lymphoid cells was investigated based on flow cytometry and whole transcriptome RNA-sequencing (RNA-Seq).^106^ In a coculture system of MitoTracker-labeled MSCs and peripheral blood mononuclear cells, MSCs mainly transferred their mitochondria to CD4^+^ T cells rather than CD8^+^ T cells or CD19^+^ B cells.^106^ Furthermore, artificially transplanted, MSC-isolated mitochondria were shown to be internalized by CD3^+^ T cells.^106^ RNA-Seq of CD3^+^ T cells with and without exogenous mitochondria showed that artificial mitochondrial transplantation increased the expression of mRNAs that are associated with T cell activation and T-regulatory (Treg) cell differentiation (FOXP3, IL2RA, CTLA4, and TGFβ1), leading to an increase in Treg cell numbers and a subsequent immunosuppressive effect.^106^ Thus, intercellular mitochondrial transfer might be a novel target for MSCs that could be exploited to mediate immunoreactions and treat immune diseases.

### Mitochondrial transfer for intercellular degradation (Table 2)

Another aspect of mitochondrial transfer is that damaged mitochondria can be delivered to other cells for degradation, reutilization, or even rescue signal transport. Davis et al.^21^ first described the transcellular degradation of mitochondria by using a virus introduced tandem fluorophore protein reporter. Damaged mitochondria in retinal ganglion cells in the optic nerve head were shown to be transferred to adjacent astrocytes and degraded by the lysosomal pathway, which revealed a new mitochondrial degradation process in retinal ganglion cells named transmitophagy, in contrast to traditional mitophagy. Interestingly, in contrast to mitochondrial transfer from healthy MSCs to macrophages observed in the repair of tissue injury,^62,63^ MSCs under oxidative stress can also extrude their partially depolarized mitochondria to macrophages via microvesicles (MVs) in a coculture system.^107^ This process is considered to be an outsourced form of mitophagy that is conducted by MSCs to ensure their survival in the presence of oxidative stress. Surprisingly, the transferred depolarized mitochondria were reutilized via mitochondrial fusion in recipient macrophages to enhance bioenergetics.^107^ Although stressed mitochondria were partially depolarized, the structure of the mitochondrial membrane was not destroyed, which provided the precondition for subsequent fusion with healthy mitochondria in recipient macrophages.^107^ Cancer cells can also export their damaged mitochondria to normal cells within their TME to adapt to different disruptions, thus favoring tumor progression. Wang et al.^91^ revealed the bidirectional transfer of mitochondria between BM-MSCs and T cell ALL (T-ALL) cells via TNTs and revealed that chemotherapy-treated T-ALL cells transferred many more mitochondria to adherent MSCs than those received from MSCs, which led to a reduction in mitochondrial ROS in T-ALL cells and enhanced their chemoresistance capacity. Moreover, ALL cells and AML cells present different adhesive capacities and mitochondrial transfer directions, which may be attributed to their different metabolic states. T-ALL cells prefer glycolysis after coculture, while AML cells tend to depend more on OXPHOS.^108^ Therefore, it is expected that ALL cells will prefer to export their stressed mitochondria to reduce intracellular ROS, while AML cells will tend to import functional mitochondria to meet their demand for OXPHOS.^91^

Mitochondrial damage-associated molecular patterns (DAMPs), such as mtDNA, *N*-formyl peptides, and mitochondrial proteins released from damaged cells, will be recognized by immune cells (neutrophils and monocytes/macrophages) and result in an immune response.^109,110^ Thus, mitochondria can be considered not only as sensors of cellular stress but also as regulators of danger signaling to alert the cell or tissue to response.^111^ Particularly, recent findings revealed that the entire mitochondria can also be released from damaged cells and act as special DAMPs to deliver danger signals for inflammatory responses^112^ or tissue injury repair.^113^ In a coculture system, mitochondria released from damaged somatic cells (CMs or endothelial cells) were engulfed and degraded by MSCs through the heme oxygenase-1 (HO-1) signaling pathway, which was followed by subsequent stimulation of mitochondrial biogenesis in MSCs.^113^ The activated mitochondria in MSCs were then donated to H_2_O_2_-stressed somatic cells to rescue the cells from apoptosis.^113^ As a critical autophagy trigger,^114,115^ ROS produced in stressed cells was revealed to be a crucial damage signal necessary to induce the HO-1-mediated anti-apoptotic response. The absence of ROS in stressed somatic cells reduced the donation of mitochondria from MSCs.^113^ Although the specific mechanisms are still unclear, such a process involving transfer of damaged mitochondria from somatic cells to MSCs for degradation as well as transfer of healthy mitochondria in MSCs to damaged cells indicated a rational direction for further investigation of the mechanisms of intercellular mitochondrial transfer.

## Mechanisms of mitochondrial transfer

To date, the molecular and signaling mechanisms of mitochondrial transfer are still obscure. Most studies have indicated that TNTs and MVs are the most popular routes of intercellular mitochondrial transport. However, the factors that trigger mitochondrial transfer and subsequent cellular activities in donor cells and recipient cells are less understood. Here, we list the evidence and the diverse molecular clues to better understand the potential mechanisms of intercellular mitochondrial transfer for future study.

### The route of intercellular mitochondrial transfer

#### Tunneling nanotubes

**Fig. 1:**
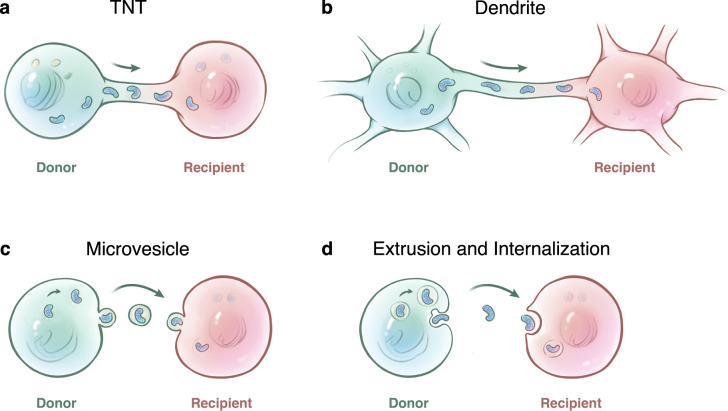
Routes of mitochondrial transfer from donor cells to recipient cells. **a** TNT is a membranous tubular protrusion that extends from the plasma membrane, with a variety of diameters between 50 and 1500 nm and lengths from several tens to hundreds of microns. TNT is the most popular route of mitochondrial transfer between the connected cells. **b** Dendrite is another form of the membranous protrusion. Some cells with dendrites (e.g., osteocytes) are connected to each other via intrinsic dendrites to form an intercellular network, which provides a highway for mitochondrial transfer. **c** Microvesicles formed by blebbing of the cellular plasma membrane were reported as another route for mitochondrial transfer. **d** Free mitochondria alone can be extruded or internalized without carriers, which provides a possible route for intercellular mitochondrial transfer

TNTs were first reported to exist between rat pheochromocytoma PC12 cells^9^ and immune cells^116^ in 2004. They are spontaneous membranous tubular protrusions that extend from the plasma membrane (PM) with various diameters between 50 and 1500 nm and are several tens to hundreds of microns in length,^117^ which allows the trafficking of various cellular components or organelles and largely facilitates long-distance cell-to-cell communication for tissue homeostasis.^13^ Among all the transported cargoes that had been described in the past, the mitochondrion appears to be the most frequently reported organelle that can be unidirectionally or bidirectionally transferred via TNTs (Fig. 1a). In addition, other TNT-like membranous protrusions, such as the intrinsic dendrites of osteocytes, were also confirmed to act as a critical route for the transfer of mitochondria within the osteocyte dendritic network (Fig. 1b).^73^ By selectively blocking the formation of TNTs via the actin-binding toxin, cytochalasin B, at nanomolar concentration (350 nM), which had negligible effects on endocytosis and phagocytosis activities, the organelle transfer between cells was dramatically reduced.^118^

**Fig. 2:**
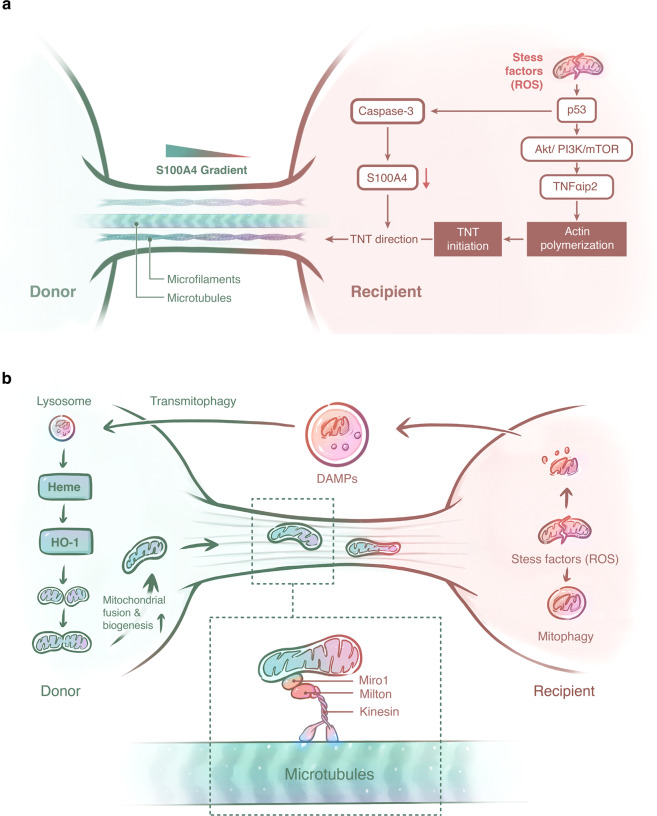
The mechanisms of mitochondrial transfer. **a** On the one hand, the generation of ROS in stressed mitochondrial recipient cells could activate p53 and its downstream Akt/PI3K/mTOR pathway, leading to the overexpression of TNFαip2, which will promote actin polymerization and TNT formation. On the other hand, the activated p53 in recipient cells could induce the activity of caspase-3 to cleave intracellular S100A4, which will generate a chemical gradient of S100A4 and contribute to the TNT growth direction from initiating cells with a low concentration of S100A4 to targeted cells with a higher concentration of S100A4. **b** In mitochondrial recipient cells, multiple stress factors will induce the generation of excess ROS, which will then trigger the fragmentation of mitochondria for mitophagy. At the same time, extra damaged mitochondria and other DAMPs will be released from the stressed cell and accepted by mitochondrial donor cells for transmitophagy. The degradation of damaged mitochondria by lysosomes in donor cells will lead to the release of heme, which will then trigger the HO-1 pathway and increase the biogenesis of mitochondria in donor cells, followed by the fusion of mitochondria. Functional mitochondria in donor cells are then transferred to stressed cells. Similar to axonal mitochondrial transport, the movement of mitochondria on microtubules within the TNT might also rely on the Miro1/Milton complex and its connection with kinesin

As we described above, various stress factors that induce mitochondrial damage could facilitate the formation of TNTs and the subsequent transfer of mitochondria, but few studies have focused on the initiation mechanism and regulation of these membranous protrusions. p53 activation was reported as an important TNT-initiating factor in response to cellular stress.^119^ In stress-exposed cells, the activation of p53 triggered the upregulation of EGFR and its downstream Akt/PI3K/mTOR pathway, leading to the overexpression of M-Sec (TNFαip2),^119^ which promoted actin polymerization and TNT formation at the cell membrane by interacting with RalA and the exocyst complex.^120^ Intriguingly, TNT formation between rotenone-injured CECs and MSCs was shown to be mediated by the upregulation of the NF-κB/TNFαip2 signaling pathway, which was activated by rotenone-induced ROS.^75^ Furthermore, we can find more clues concerning the initiation process of TNT formation because p53 associates closely with ROS,^121,122^ and the activation of p53 could be triggered by ROS produced by oxidative stress.^123^ In addition to the initiation of TNT formation, p53 activation also increases the activity of caspase-3 to cleave intracellular S100A4,^124^ a member of the calcium-binding S100 protein family.^125^ It was reported that the chemical gradient of S100A4 contributes to TNT growth from initiating cells with a low concentration of S100A4 to targeted cells with a higher concentration of S100A4.^124^ Together with these evidences, a schematic diagram illustrating potential mechanisms of TNT formation between cells is presented in Fig. 2a.

In an in vivo study of ALI, Islam et al.^11^ emphasized the positive effect of connexin 43 (Cx43), a transmembrane gap junction protein, on mitochondrial transfer by stabilizing the attachment of BMSCs to LPS-treated alveolar epithelial cells as well as promoting the formation of TNTs and MVs. However, the formation of TNTs and MVs was inhibited in Cx43-mutated BMSCs, which potentially resulted from the failure of attachment between BMSCs and alveoli. Consequently, the subsequent mitochondrial transfer and lung injury rescue were also attenuated. Nevertheless, some other studies also reported the involvement of Cx43 in TNT formation.^85,126,127^ Osswald et al.^85^ verified that mitochondria traveled quickly within the tumor membrane microtubule network, and that Cx43 was frequently located at the intersection area of two different microtubules, which facilitated calcium propagation across tumor microtubules. The knockdown of Cx43 reduced synchronicity of intercellular calcium waves and the proportion of astrocytoma cells with multiple microtubules, which indicated the role of Cx43 in stabilization of intercellular membrane microtubules in tumor cells. In addition, Cx43 was also reported to be abundant in the osteocyte dendritic network to promote the osteocyte coupling and survival,^128^ indicating that Cx43 might also contribute to the transfer of mitochondria between osteocytes by strengthening intercellular contacts. Although the mechanisms underlying the role of gap junction proteins in intercellular mitochondrial transfer require further investigation, it is possible that Cx43 contributes to the connection between TNTs and the anchored membranous structure.

As reported, the intercellular movement of mitochondria through TNTs requires the transport carrier known as Miro1, which is a calcium-sensitive Rho-GTPase in the outer mitochondrial membrane.^31,32,60,69^ In neurons, Miro1 acts as a mitochondria-loaded vehicle that interacts with mitofusion1/2 and combines with the kinesin-1 molecular motor through the Milton adaptor protein (TRAK1/2 and OIP106/98) to form a complex, thus enabling the shuttling of mitochondria along microtubules.^129,130^ Ahmad et al.^69^ revealed the effect of Miro1 on promoting TNT-mediated intercellular mitochondrial transfer from MSCs to stressed epithelial cells. The overexpression of Miro1 in MSCs enhanced the transfer of mitochondria and the rescue of injured epithelial cells, while Miro1 knockdown in MSCs led to the inhibition of mitochondrial transfer and a reduction in rescue efficiency. Intriguingly, compared with BM-MSCs, iPSCs showed higher efficiency in mitochondrial donation as well as increased rescue of anthracycline-induced cardiomyopathy due to the higher expression of Miro1.^60^ Babenko et al.^31^ also found a close relationship between Miro1 and mitochondrial transfer, as evidenced by the elevation of Miro1 levels in MSCs cocultured with neuron cells, and the mitochondrial transfer efficacy was enhanced in MSCs with Miro1 overexpression during their administration to rats with stroke.^32^ Thus, it is possible that the transfer of mitochondria between TNT-connected cells shares similar mechanisms with Miro1-mediated axonal transport of mitochondria in neurons (Fig. 2b).

#### Microvesicles

Extracellular MVs are important carriers for intercellular communication.^7^ They can be divided into several types with diverse sizes according to their origins, which mainly include exosomes (30–100 nm), MVs (100 nm to 1 µm), and apoptotic bodies (>1 µm).^7^ As they are limited in size, exosomes that derived from endosomal cell membranes can only load small proteins, lipids, and RNAs,^131,132^ as well as mtDNA.^133^ MVs formed by blebbing of the cellular PM can contain the whole organelle due to their larger diameter and were reported to participate in the transfer of mitochondria and mtDNA (Fig. 1c).^10–12,62,63,107,134^ Islam et al.^11^ reported that BMSCs donate their functional mitochondria to alveolar epithelial cells not only through TNTs but also through MVs in a Cx43-dependent manner. In addition, MVs also participate in the transmitophagy of damaged retinal ganglion cells^21^ and stressed MSCs,^107^ resulting in self-protection and reutilization of depolarized mitochondria, respectively.

#### Extrusion and internalization of free mitochondria

As depicted above, the transfer of mitochondria from donor cells to recipient cells relies on membranous carriers, such as TNTs, dendrites, and MVs, in most cases. However, some studies have also reported that free mitochondria or mitochondrial components alone can be extruded or internalized without carriers. Although the evidence for the physiological extrusion and uptake of intact mitochondria from donor cells into recipient cells is relatively weak, the transmembrane motility of free mitochondria also provides a possibility for intercellular mitochondrial transfer (Fig. 1d).

The extrusion of mitochondria mostly occurs as a means of mitochondrial quality control^135,136^ or danger signal transduction^112,137^ when the cells are under stress. For example, HeLa cells extruded their fragmented mitochondria for extracellular mitoptosis under ROS stress.^135^ Nakajima et al.^136^ described that PM-originated cytoplasmic vacuoles engulfed damaged mitochondria and then extruded them from tumor necrosis factor-α (TNFα)-induced dying cells by fusing with the PM again in a caspase-dependent manner. Interestingly, intact actin and tubulin cytoskeletons were also revealed to be necessary for membrane blebbing and mitochondrial extrusion as the destabilization of actin or tubulin inhibited the formation of cytoplasmic vacuoles. In some cases, free mitochondria extruded from stressed cells were also indicated as a special danger signal to provoke inflammatory responses.^112,136^ In Fas-associated protein with death domain-deficient Jurkat (human T lymphoblastic leukemia) and L929 (murine fibroblast) cells treated with TNF-α, mitochondrial fission and extracellular release of intact mitochondria were detected.^112^ Further in vitro studies suggested that the purified mitochondria released from necroptotic cells could be engulfed by human macrophages and dendritic cells, leading to the modulation of cytokine production from macrophages and induction of dendritic cell maturation.^112^ In another study, Boudreau et al.^137^ revealed that functional mitochondria could be released from activated platelets as both membrane-encapsulated microparticles and free organelles. In addition to in vitro studies, an in vivo study conducted by Boudreau et al.^137^ revealed that the extracellular mitochondria intravenously injected into mice were found to associate with neutrophils and prompt neutrophil adhesion to the vascular wall, resulting in the activation of neutrophils and inflammatory responses.

The internalization of free mitochondria was first described by Clark and Shay,^138^ who termed this phenomenon “mitochondrial transformation,” in 1982. In their study, chloramphenicol (CAP)- and efrapeptin (EF)-sensitive mammalian cells were able to internalize free mitochondria purified from CAP- and EF-resistant fibroblasts in a coincubation environment, resulting in an increase in their survival rate under antibiotic exposure.^138^ This research suggests that the effect of mitochondrial transformation could serve as a therapeutic target. By transplanting isolated healthy mitochondria, which were thought to be internalized by recipient cells via macropinocytosis,^139,140^ the OXPHOS capacity, proliferation, and bioenergetics of recipient cells could be restored in vitro.^35,86,93,139–141^ Recent studies demonstrated that free mitochondrial internalization relies on the integrity of outer mitochondrial membrane and the fusion proteins (such as syncytin-1 and syncytin-2) on it, which may act as ligands in the interactions between mitochondria and recipient cells.^86,140^ In addition to in vitro studies, some in vivo studies also indicated the effect of mitochondrial transplantation on tissue damage rescue.^37,56,142,143^ Notably, McCully’s team conducted a series of in vivo and clinical studies to examine the cardioprotective effect of mitochondrial transplantation on myocardial ischemia–reperfusion injury.^56,142,143^ Healthy autologous mitochondria isolated from non-ischemic skeletal muscle of rabbits were injected directly into the ischemic zone of the heart, where they could be internalized by damaged CMs, resulting in a reduction in the infarct size and the improvement of postinfarct cardiac function.^56^ Furthermore, they reported the first clinical application of mitochondrial autotransplantation for myocardial recovery in pediatric patients who were supported by extracorporeal membrane oxygenation (ECMO) due to ischemia–reperfusion injury.^143^ Although some issues concerning the dose and route of mitochondrial transplantation still need to be optimized, their primary results were encouraging, as four of the five patients showed improvement in their ventricular function and were successfully separated from ECMO support.^143^ To date, the mechanism of free mitochondrial uptake has not been fully clarified, but the transplantation of free functional mitochondria appears to be a potential therapeutic strategy for the treatment of tissue damage.

### Trigger signals

As the evidence accumulated, researchers realized that stress signals produced by the recipient cells due to mitochondrial damage or intracellular oxidative stress were probably transferred to donor cells for mitochondrial transfer initialization (Fig. 2b). In addition to DAMPs and the whole damaged mitochondria that we referred to above,^109,110,112,113^ some other molecules and their corresponding pathways were also reported to catalyze this process. During OXPHOS in normal mitochondria, a small fraction of the electrons will leak out from complexes I and III, generating extra ROS by reacting with O_2_.^17^ Under physiological conditions, these byproducts can be decomposed by antioxidant enzymes such as superoxide dismutase (SOD), catalase (CAT), or glutathione peroxidase (GPx) to reduce the detrimental effect of ROS as well as control cellular homeostasis.^17^ However, under different pathological conditions, cells suffering from either ischemia–hypoxia or chemical hazards that disrupt mitochondria function will produce excess ROS, which cannot be efficiently diminished by these antioxidant enzymes, thus leading to oxidative damage. In high energy-consuming cells, which are frequently reported to act as mitochondrial recipient cells, stress-induced ROS tend to accumulate and to trigger intercellular mitochondrial rescue.^69,89,113^ Conversely, MSCs, which generally act as mitochondrial donor cells, maintain their mitochondria in a dormant state and prefer glycolysis because of their low energy demands,^144^ which undoubtedly decreases the risk of ROS production. In addition, MSCs express high levels of active SOD, CAT, and GPx to control the level of ROS.^145^ During stem cell differentiation, the cellular metabolism favors OXPHOS, which is accompanied by enhanced mitochondrial biogenesis and the reshaping of the morphology of mitochondria from fragmented to elongated to meet the higher energy demands.^146–149^

Under stress, increased ROS was shown to induce mitochondrial fission and perinuclear clustering of the resulting punctate mitochondria for subsequent mitochondrial extrusion and extracellular mitoptosis.^135^ The degradation of damaged mitochondria, also called mitophagy, requires prior mitochondrial fission to facilitate engulfment of fragmented mitochondria by autophagosomes.^150^ Intriguingly, the transfer of damaged mitochondria from impaired somatic cells pretreated with the ROS scavenger (*N*-acetyl-l-cysteine, NAC) to MSCs was significantly attenuated.^113^ The activation of HO-1 and mitochondrial biogenesis in MSCs, as well as the donation of MSC mitochondria to somatic cells, were all inhibited.^113^ As mitochondria are enriched in heme-containing proteins, a reasonable scenario was proposed in which the ROS-driven transmitophagy of stressed mitochondria derived from recipient somatic cells led to the release of heme in MSCs, which triggered the HO-1 pathway in MSCs (Fig. 2b).^113^ Consistent with the fact that HO-1 is known to increase mitochondrial biogenesis,^151,152^ the activation of HO-1 elevated the expression of proliferator-activated receptor gamma coactivator-1α and mitochondrial transcription factor A in MSCs, which probably promoted mitochondrial fusion for subsequent mitochondrial donation to aid in rescuing the stressed somatic cells (Fig. 2b).^113^ In addition, a recent study also confirmed the effect of ROS on triggering mitochondrial transfer from hematopoietic stem cells (HSCs) to BM-MSCs.^153^ In detail, the accumulation of ROS in HSCs induced by Gram-negative bacterial infection activated PI3K signaling and thus facilitated mitochondrial transfer from BM-MSCs to HSCs in a Cx43-dependent manner.^153^ Coincidentally, as described above, the activation of Akt/PI3K/mTOR pathway by p53 was also associated with the formation of TNTs, leading to the overexpression of TNFαip2 (Fig. 2a). Regarding the close relationship between ROS and p53, ROS in stressed cells is probably the initiator of mitochondrial transfer, and downstream PI3K might be the critical mediator involved in promoting cell–cell contact and thus facilitate the formation of transfer route.

CD38, a multifunctional transmembrane glycoprotein, is known as a catalyst for the synthesis of calcium messenger cyclic ADP-ribose^154^ and nicotinic acid–adenine dinucleotide phosphate^155^ from nicotinamide adenine dinucleotide (NAD^+^) and nicotinamide adenine dinucleotide phosphate (NADP^+^), respectively. These reactive metabolites are essential for intracellular calcium mobilization. Recently, CD38 was shown to participate in the process of mitochondrial transfer in two different models.^12,84^ Intriguingly, CD38 in donor astrocytes promoted the transfer of mitochondria to adjacent neurons via MVs,^12^ whereas CD38 in recipient multiple myeloma cells drove the acquisition of mitochondria from neighboring BMSCs via TNTs.^84^ On the one hand, the expression of CD38 in astrocytes is mediated by neuron-released glutamate in the coculture system,^156^ and excessive glutamate also stimulates the generation of ROS in neurons;^157^ thus, it is probable that excitotoxic glutamate in ischemic neurons might be a potential trigger for mitochondrial transfer from adjacent astrocytes. On the other hand, CD38 was also known to facilitate cell adhesion,^158^ and CD38 expression in multiple myeloma cells was positively correlated with TNT anchor points in cocultured BMSCs,^84^ indicating that CD38 expression is linked to nanotube attachment. Several studies have demonstrated that LPS, as a stress factor, can induce mitochondrial transfer^11,59,62,63^ and LPS can also upregulate the expression of CD38.^159^ Thus, CD38 may also play a role in the initiation of mitochondrial transfer, although questions still remain regarding the specific function of CD38 in this process.

### ER mediates mitochondrial transfer

**Fig. 3:**
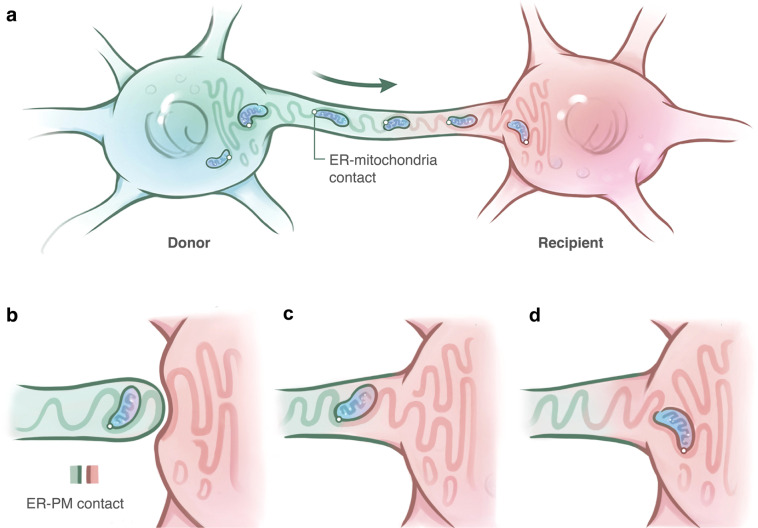
The hypothetical role of ER in mediating mitochondrial transfer. **a** Mitochondria are transferred along with the ER tubular network within the dendrites of osteocytes via ER–mitochondria contact. **b** The dynamic transfer of mitochondria between osteocytes requires dendrite-mediated cell–cell contacts. The mitochondrion is delivered to the terminus of the dendrite, where the ER may contact with the PM. The arrival of mitochondria at the ER–PM contact might change the lipid metabolism of the PM temporarily. **c** The ER in the donor osteocyte may fuse with the ER extension from the recipient osteocyte to facilitate the docking of mitochondria to the recipient cell. **d** Mitochondria from donor osteocytes are transferred to recipient osteocytes and continue moving along with the ER tubular network

ER interacts closely with mitochondria and has been revealed to play a critical role in regulating mitochondrial biogenesis via ER–mitochondria contact.^160^ ER–mitochondria contact also functions to coordinate multiple processes in these two organelles, including Ca^2+^ signaling, lipid synthesis, and intracellular mitochondrial trafficking.^160^ As reported, Mfn2 was necessary for axonal mitochondrial movement, during which it interacted with the Miro/Milton complex in microtubule-based transport systems.^129^ The pause time increased and the movement velocities decreased for the axonal transport of mitochondria in Mfn2-deficient neurons.^129^ Considering the role of Mfn2 in tethering mitochondria to the ER, an appealing possibility is that Mfn2 regulates axonal mitochondrial transport via ER–mitochondria contact. Recently, our group demonstrated the dynamic transfer of mitochondria between osteocytes along the tubulin track of their dendrites (Fig. 3a), and this process requires osteocyte dendrite-mediated cell–cell contacts (Fig. 3b).^73^ Furthermore, the transfer of mitochondria was dynamically associated with the ER (Fig. 3a).^73^ The inhibition of Mfn2 or vesicle-associated membrane protein B, another ER–mitochondria tethering protein, significantly inhibited the transfer of mitochondria within the osteocyte dendritic network, which verified the critical role of ER–mitochondria contact in mitochondrial transfer.^73^

Both microfilaments and microtubules are major components within tubular membranous structures (including TNTs and dendrites),^13^ which are the most popular routes for intercellular mitochondrial transfer. TNT formation relies on the polymerization of actin, which implies the extension of microfilaments. Mitochondrial movement, either within the axons of neurons^129,130^ or in TNTs,^31,32,69^ relies on Miro1 via its interaction with the motor protein (kinesin-1), which enables the shuttling of mitochondria along with microtubules. Another study revealed that ER sliding only occurred on stable acetylated microtubules, and mitochondria preferentially localized to these regions as well,^161^ and the contact sites between the ER and mitochondria were still maintained even during their dynamic morphological processes.^161^ Furthermore, the distribution of ER within cells also relies on microtubule alignment.^162–164^ It is possible that mitochondria form a complex together with the ER and microtubules during the transfer process in tubular membranous structures, and microfilaments that extend the cytoskeleton provide the structural foundation for mitochondrial trafficking. Therefore, it is possible that microtubules, microfilaments, and ER–mitochondria contacts may coordinate concurrently to facilitate the transfer of mitochondria between cells based on the route of tubular membranous structures. However, the route of mitochondrial transfer varies among different cells, and different transfer routes might imply different mechanisms. It remains unclear whether microfilaments, microtubules, and ER–mitochondria contact are indispensable elements for mitochondrial trafficking via other routes.

## Perspectives and applications

Although mitochondrial transfer has been broadly revealed to have multiple modes and appears to be a novel aspect of the biological responses involved in tissue homeostasis and development, some critical questions still remain to be answered.

First, the route of mitochondrial transfer varies among different systems. It remains unclear whether the route is specifically dependent on the donor or recipient cell types. Furthermore, the process of the initiation of TNTs is also unclear. In our diagram (Fig. 2a), the signaling mechanisms of TNT initiation from recipient cells were shown by several studies.^75,119,120,123,124^ While other studies demonstrated that TNTs were derived from donor cells,^11,60,62^ the initiation mechanisms were not clearly investigated. Intriguingly, in cells with dendrites, such as osteocytes and astrocytes, the self-developed dendrites that extend from the PM can also be deemed as a special form of TNTs and facilitate the transport of mitochondria within their dendritic networks. However, how mitochondria break through the membrane barriers and are delivered to the membranous contact points between two cells is not fully understood. Although the gap junction protein Cx43 was reported to support mitochondrial transfer, it is unlikely that mitochondria can directly pass through such narrow channels. Cx43 may only function as a stabilizer to adhere to the membrane structures of two cells. Our group verified the role of ER–mitochondria contact in the transfer of mitochondria from healthy to stressed osteocytes via dendrites (Fig. 3a).^73^ Similar to the dendritic extension of the PM, it is probable that the ER also regulates intercellular mitochondrial transfer in TNT-mediated models. However, many detailed mechanisms are still unclear. For example, how does the ER drive the movement of mitochondria on microtubules? What happens to ER–mitochondria contact before mitochondria is delivered to the recipient cell at the cell junction? Does the ER play a role in the breakthrough of the membrane barrier at the cell contact point and facilitate the transition of mitochondria via the microtubules of donor and recipient cells? When considering these issues, it should not be ignored that the ER tubular network also makes close contact with the PM (Fig. 3b) and ER–PM contact plays a critical role in Ca^2+^ signaling and lipid metabolism.^165,166^ Thus, it is highly probable that the ER is the crucial mediator of the delivery of mitochondria from the cytoplasm to the PM by tethering with mitochondria to its tubular network and facilitating the subsequent breakthrough of membrane barriers via ER–PM contact (Fig. 3b–d). These processes will inevitably involve changes in intracellular ion homeostasis (especially Ca^2+^) and the lipid metabolism at the membranous anchor points among the mitochondria, ER, and plasma membrane, while the particular mechanisms are still obscure.

Second, Mahrouf-Yorgov et al.^113^ demonstrated that the degradation of stressed mitochondria derived from injured somatic cells was required for the activation of HO-1 pathway in healthy MSCs and therefore the initiation of energetic mitochondrial donation to stressed somatic cells. In mitochondria-stressed cells, damaged mitochondria undergo fragmentation prior to selective mitophagy.^150,167^ It has been shown that ER–mitochondria contact coordinates mitochondrial division.^168,169^ Thus, it is tempting to speculate that ER also participates in the intercellular degradation of stressed mitochondria in an unknown manner. If so, questions can be posed: is the ER of stressed cells also transferred along with damaged mitochondria on microtubules? It also remains unclear whether this kind of “transmitophagy” always coexists with intrinsic mitophagy as another common method of mitochondrial quality control, or if it only occurs when the damage to mitochondria surpasses a certain threshold so that intrinsic mitophagy is not adequate to eliminate all the stressed mitochondria. In such conditions, intercellular mitochondrial donation ideally compensates for the OXPHOS deficiency in stressed cells and therefore revitalizes the damaged tissue. Nevertheless, further studies still need to be conducted to unravel the mysteries of intercellular mitochondrial degradation and its role in mitochondrial quality control.

Third, mitochondrial motility normally corresponds to the changes in mitochondrial biogenesis, which results in the changes in intracellular respiratory metabolism. Although the elevation of mitochondrial biogenesis and mitochondrial fusion was observed in donor MSCs before mitochondrial donation,^113^ it is not clear whether such mitochondrial biogenesis alteration is compulsory and whether the transferred mitochondria need to be further processed in recipient cells. Furthermore, we noticed that functional mitochondrial transfer could occur in a unidirectional or bidirectional manner, but little is known regarding the reasons for such directional differences in different intercellular models. Efforts should be made in the future to distinguish whether the intrinsic difference in respiratory status between the mitochondrial donor and recipient cells is responsible for the mitochondrial donation capacity variance.

**Fig. 4:**
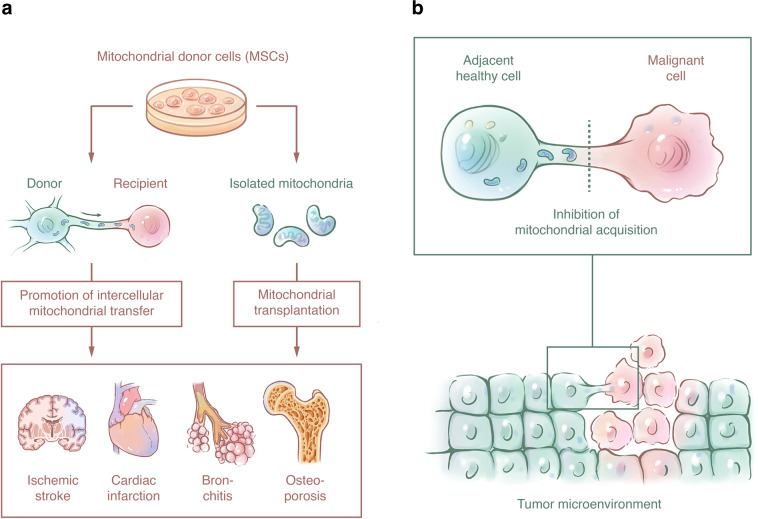
The potential therapeutic applications of mitochondrial transfer. **a** Promoting the transfer of healthy mitochondria from mitochondrial donor cells to stressed cells or isolating healthy mitochondria from donor cells and transplanting them to stressed tissues can be developed as prospective therapeutic strategies for the treatment of diseases caused by dysfunctional mitochondria (e.g., ischemic stroke, cardiac infarction, bronchitis, and osteoporosis). **b** Inhibiting the acquisition of functional mitochondria from adjacent healthy cells by malignant cells within the tumor microenvironment can also serve as a novel target to combat cancer progression and drug resistance

As mitochondrial impairment is responsible for ischemic–hypoxic tissue damage and a number of inherited mitochondrial diseases, the mitochondria-targeted treatment appears to be a promising option to reduce mitochondrial deficiency. However, the efficacy of current therapy strategies that mainly focus on mitochondrial respiratory chain repair and mtDNA modification is still limited.^170,171^ Given that undifferentiated stem cells are the most common candidates for mitochondrial donation, which probably contributes to the therapeutic effect of stem cells, investigations of the mechanisms that enhance the transfer efficacy of mitochondria, either by promoting the formation of transfer routes or reinforcing the factors that facilitate mitochondrial motility (Miro1 and ER–mitochondria contact), will lead to the development of a new era for the treatment of mitochondrial dysfunction (Fig. 4a). Considering that spontaneous mitochondrial transfer occurs under physiological conditions and the potential roles it plays in maintaining tissue homeostasis, encouraging the transfer of mitochondria between cellular networks appears to be a possible treatment for degenerative diseases during aging, such as AD and osteoporosis. In addition, the extrusion and internalization of free mitochondria make it possible to perform direct mitochondrial isolation and transplantation for therapeutic purposes (Fig. 4a),^56,142,172^ which was reviewed in detail by Caicedo et al.^173^ However, several concerns still remain regarding the species origin and ideal cell types for mitochondrial donation, as well as the potential ethical problems.^173^ Some progress has been made to enhance the efficacy of the transfer of isolated mitochondria into recipient cells.^174,175^ Maeda et al.^175^ innovatively combined mitochondria with the transactivator of transcription (TAT) peptide of human immunodeficiency virus and dextran, which greatly enhanced the cellular incorporation of isolated mitochondria and improved the rescue effect of mitochondrial replenishment in damaged CMs. In the TAT–dextran complex, the TAT peptide facilitates the engulfment of mitochondria by recipient cells, and dextran stabilizes the combination with the outer mitochondrial membrane. Further efforts are still needed to optimize the process of mitochondrial isolation and artificial transplantation to improve the purity and efficacy of mitochondrial therapy.

Finally, as increasing evidence has illustrated the protective effect of cell-to-cell mitochondrial trafficking within the TME on the survival of malignant cells, inhibiting the intercellular acquisition by malignant cells of functional mitochondria from adjacent healthy cells can also serve as a novel strategy to combat cancer progression and drug resistance (Fig. 4b). For example, CD38 has been reported to play an important role in promoting intercellular mitochondrial transfer. A new study indicated that daratumumab, an anti-CD38 monoclonal antibody, could inhibit AML disease progression via a mechanism involving blocking mitochondrial transfer from MSCs to AML blasts.^176^ As malignant tumors develop as part of a high-metabolic and invasive complex, future studies are required to investigate the profound effect of mitochondrial acquisition on the respiratory metabolism of cancer cells and to explore a valid approach for the inhibition of mitochondrial transfer in the TME with minimal side effects.

## Conclusions

As discussed in this review, the intercellular transfer of mitochondria is a universal biological event that occurs with or without stress factors for energy synchronization, although many of the details of this process are still largely unclear. Therapeutically, it would be lucrative to exploit the new insights into its mechanisms to selectively catalyze or interrupt this kind of mitochondrial communication for tissue revitalization and homeostasis as well as tumor suppression. Further investigations are imperative to clarify gaps in knowledge, including the levels of stress factors that induce mitochondrial transfer, the formation mechanisms of intercellular connections in different transfer models, alteration of mitochondrial biogenesis in both donor and recipient cells and the role the ER plays in cell-to-cell mitochondrial trafficking. Moreover, stem cell transplantation and direct mitochondrial transplantation have been demonstrated to be effective in rescuing tissue damage in a mitochondrial transfer-associated manner. Although there are still many critical barriers to overcome, a better understanding of optimal conditions for mitochondrial transfer could lead to a breakthrough in translation of mitochondrial transfer and transplantation in the clinic.
